# *biosigner*: A New Method for the Discovery of Significant Molecular Signatures from Omics Data

**DOI:** 10.3389/fmolb.2016.00026

**Published:** 2016-06-21

**Authors:** Philippe Rinaudo, Samia Boudah, Christophe Junot, Etienne A. Thévenot

**Affiliations:** ^1^CEA, LIST, Laboratory for Data Analysis and Systems' Intelligence, MetaboHUBGif-sur-Yvette, France; ^2^Laboratoire d'Etude du Métabolisme des Médicaments, DSV/iBiTec-S/SPI, MetaboHUB, CEA-SaclayGif-sur-Yvette, France

**Keywords:** feature selection, biomarker, molecular signature, omics data, partial least squares, support vector machine, random forest

## Abstract

High-throughput technologies such as transcriptomics, proteomics, and metabolomics show great promise for the discovery of biomarkers for diagnosis and prognosis. Selection of the most promising candidates between the initial untargeted step and the subsequent validation phases is critical within the pipeline leading to clinical tests. Several statistical and data mining methods have been described for feature selection: in particular, wrapper approaches iteratively assess the performance of the classifier on distinct subsets of variables. Current wrappers, however, do not estimate the significance of the selected features. We therefore developed a new methodology to find the smallest feature subset which significantly contributes to the model performance, by using a combination of resampling, ranking of variable importance, significance assessment by permutation of the feature values in the test subsets, and half-interval search. We wrapped our *biosigner* algorithm around three reference binary classifiers (Partial Least Squares—Discriminant Analysis, Random Forest, and Support Vector Machines) which have been shown to achieve specific performances depending on the structure of the dataset. By using three real biological and clinical metabolomics and transcriptomics datasets (containing up to 7000 features), complementary signatures were obtained in a few minutes, generally providing higher prediction accuracies than the initial full model. Comparison with alternative feature selection approaches further indicated that our method provides signatures of restricted size and high stability. Finally, by using our methodology to seek metabolites discriminating type 1 from type 2 diabetic patients, several features were selected, including a fragment from the taurochenodeoxycholic bile acid. Our methodology, implemented in the *biosigner* R/Bioconductor package and Galaxy/Workflow4metabolomics module, should be of interest for both experimenters and statisticians to identify robust molecular signatures from large omics datasets in the process of developing new diagnostics.

## 1. Introduction

High-throughput, non-targeted, technologies such as transcriptomics, proteomics, and metabolomics, show great promise for the discovery of molecular markers which allow to efficiently discriminate between biological or clinical conditions of interest (e.g., disease vs. control states; Nicholson, 2006; van 't Veer and Bernards, 2008; Boja et al., 2011). In particular, metabolomics, by focusing on the end-product of biochemical reactions, has a strong potential for phenotype characterization and biomarker discovery (Holmes et al., 2008; Zhang et al., 2015). Recent studies have described candidate biomarkers for the diagnosis or prognosis of many diseases, including diabetes (Wang et al., 2011), kidney diseases (Rowe et al., 2013; Zhao, 2013; Posada-Ayala et al., 2014), cancer (Chen et al., 2011; Armitage and Barbas, 2014), and neurodegenerative diseases (Graham et al., 2013; Mapstone et al., 2014).

Powerful statistical and data mining approaches have been developed to learn classification rules from omics datasets despite the high feature over sample ratio and the large proportion of correlated features (Trygg et al., 2007; Scott et al., 2013; Tarca et al., 2013). Such approaches include Support Vector Machines (SVM; Boser et al., 1992), Partial Least Square—Discriminant Analysis (PLS-DA; Wold et al., 2001; Barker and Rayens, 2003), and Random Forest (Breiman, 2001), which have been widely used in transcriptomics, proteomics, and metabolomics (Brown et al., 2000; Diaz-Uriarte and Alvarez de Andres, 2006; Madsen et al., 2010; Robotti et al., 2014). Although these models can achieve good predictions accuracies, the excess of features in the training dataset increases both the risk of overfitting and the prediction variability. In addition, in the context of biomarker discovery and clinical diagnostic, selection of a restricted list of candidate markers is mandatory before entering the subsequent qualification/verification phases (Baker, 2005; Rifai et al., 2006; Keating and Cambrosio, 2012).

Since the comprehensive analysis of all 2^*p*^ combinations of *p* features is not computationally tractable for large omics datasets, several statistical and data mining techniques for feature selection have been described with the common goal of extracting a restricted list of variables (i.e., a molecular signature) still providing high performance of the classifier (Guyon and Elisseeff, 2003; Saeys et al., 2007). One strategy consists in filtering the features before building the classifier (Golub et al., 1999). In such *filter* techniques, features are ranked according to a univariate (e.g., *p*-value from hypothesis testing of median differences between the two classes) or a multivariate metric (e.g., Variable Importance in Projection, VIP, from PLS-DA), and a threshold is applied. Filter methods are fast; however, since the selection is performed before the final model is built, the selected features may not be optimal for the classifier performance. In addition, the choice of the threshold may be subjective, and the size of the signature may be large. A second type of methods combines feature selection and model construction in a single step: by including a penalization constraint within the algorithm building the classifier, the *embedded* approaches limit the number of features with non-zero coefficients in the final model (e.g., Lasso, Tibshirani, 1996, Elastic Net, Zou and Hastie, 2005, and sparse PLS, Chun and Keles, 2010). Such strategies are computationally efficient but the signature may be large and subject to substantial variation upon repetition of the algorithm (instability). Moreover, only one type of classifier is used, whereas several studies have shown that best classification performances are obtained by distinct models depending on the structure of the dataset (Guo et al., 2010; Tarca et al., 2013; Determan, 2015). Therefore, a third category of approaches, called *wrapper* methods, are of interest because they can be applied to any classifier, and take into account the specificities of the classifier in the process of feature selection (Kohavi and John, 1997).

The *wrapper* feature selection methods (e.g., Recursive Feature Elimination, RFE, applied to SVM; Guyon et al., 2002) iteratively (i) select groups of features which still provide a good classification accuracy, and (ii) re-build the model on the data subset. Several heuristics have been described to find optimal combination of features (either *deterministic*, such as forward and backward selection of individual or groups of variables, or *stochastic*, such as genetic algorithms or simulated annealing; Kuhn and Johnson, 2013). A limitation of current wrapper methods is that the selection criterion is based on the classifier performance only: the added-value of including a particular group of features instead of noise into the model (which we call the feature subset *significance* hereafter) is not evaluated. Here, we therefore propose a new wrapper algorithm based on random permutation of feature intensities in test subsets obtained by resampling, to assess the significance of the features on the model performance. We wrapped our algorithm around three classifiers, namely PLS-DA, Random Forest, and SVM, and applied our feature selection approach to four real metabolomics and transcriptomics datasets, including one unpublished clinical LC-HRMS analysis of plasma samples from diabetic patients. We show that restricted, complementary, and stable molecular signatures are obtained, and that the corresponding models have high prediction accuracies.

## 2. Theory

**Figure 1:**
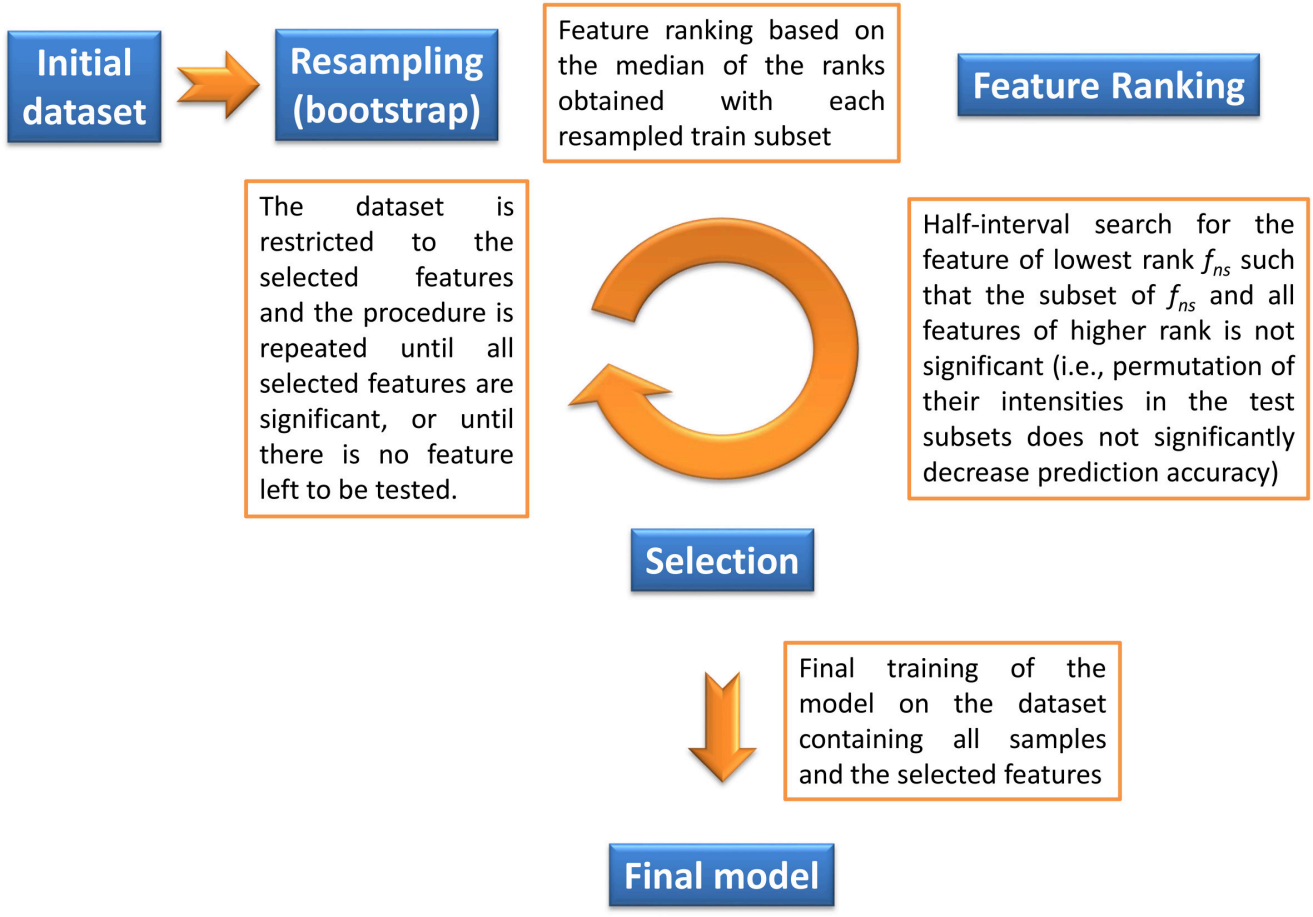
**Description of the *biosigner* algorithm for feature selection (see Section 2 for details)**. The algorithm is wrapped around the PLS-DA, Random Forest, and SVM binary classifiers.

**Figure 2:**
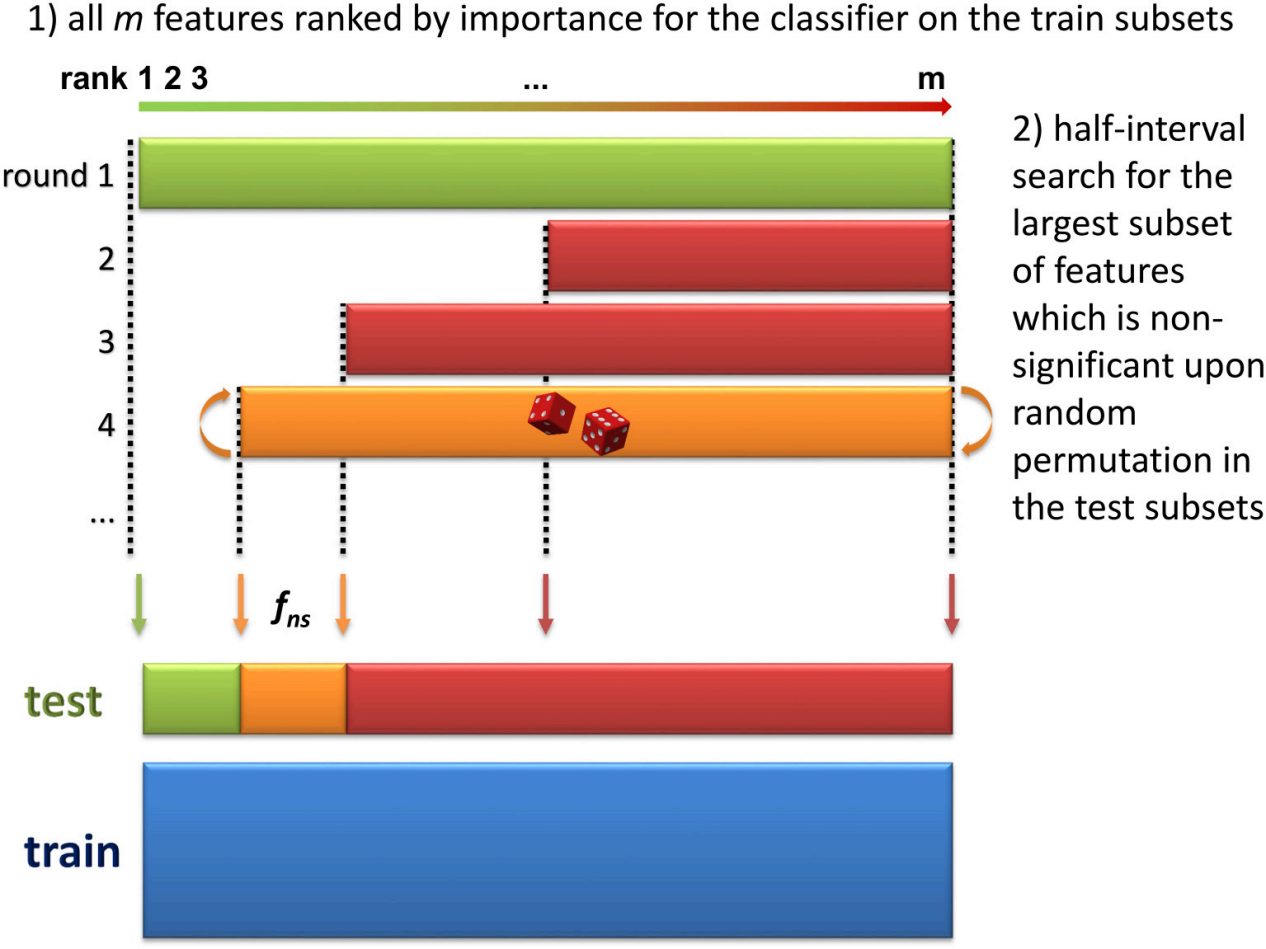
**Determination of the largest non-significant subset of features (step 3 of the *biosigner* algorithm)**. During the two first steps of the algorithm, the dataset has been split into train and test subsets by resampling, and features have been ranked according to their aggregated importance for the classifier built on the train subsets (the lower the rank, the higher the importance). In this third step, half-interval search is used to find the feature of lowest rank, *f*_*ns*_ such that the subset *S*_*f*_*ns*__ of all features with ranks ≥*rank*(*f*_*ns*_) is not significant (i.e., random permutation of all *S*_*f*_*ns*__ feature intensities in the test subsets does not significantly decrease the prediction accuracy of the model). In this example, the permutation of all features (green, 1st iteration) significantly reduced model accuracy, but not the permutation of the 50% features with highest ranks (red, 2nd iteration), nor the 75% of features with highest ranks (red, 3rd iteration).

The objective of our method is to find the significant feature subset necessary for a classifier to optimally discriminate between two classes. Given a machine learning methodology, our algorithm thus provides both the molecular signature (i.e., the significant feature subset) and the trained classifier, which can subsequently be used for prediction on new datasets. Feature selection is based on a backward procedure in which significance of each feature subset is estimated by random permutation of the intensities. The dataset is then restricted to the significant feature subset, and the whole procedure is performed iteratively until, for a given round, all candidate features are found significant (in this case the signature consists of these features), or until there is no feature left to be tested (in this case the signature is empty). The algorithm thus consists of three steps (Algorithm 1 and Figure 1):

1. **Bootstrap resampling**. A *boot* number of subsets (default is 50) are obtained by bootstrapping. Each subset consists of a training set (*train*_*k*, 1 ≤ *k* ≤ *boot*_) and the inferred test set (*test*_*k*_). On each *train*_*k*_ set, a model (*model*_*k*_) is then trained. Note that no other model needs to be built up to step 4, thus reducing the computation burden. Each *model*_*k*_ is evaluated on the *test*_*k*_, and the balanced prediction accuracy is computed (*accuracy*_*k*_).
2. **Feature ranking**. For each *model*_*k*_, the features are ranked according to a metric *rank*_*k*_ (the default metric is variable importance in projection, VIP, for PLS-DA, Wold et al., 2001, variable importance for Random Forest, Breiman, 2001, and squared weights for SVM, Guyon et al., 2002). Finally, the *rank*_*k*_ are aggregated by computing the median to obtain the final ranking:
  (1) $$\begin{array}{l} rank=RANK\left(MEDIAN_{1\leq k\leq boot}\left(rank_{k}\right)\right) \end{array}$$
  where *RANK* and *MEDIAN* are the usual ranking and median functions.
3. **Selection of significant features**. The objective of this step is to discard all non-significant features from the dataset. The method consists in finding the largest non-significant feature subset *S*_*f*_ = {*g*|*rank*(*g*) ≥ *rank*(*f*)} (or, equivalently, the feature *f*_*ns*_ of lowest rank such that *S*_*f*_*ns*__ is not significant). A half-interval search algorithm is used to find *f*_*ns*_: for a given *f*, the significance of *S*_*f*_ is estimated by randomly permuting all *S*_*f*_ feature intensities in the *test*_*k*_ subsets (Figure 2), and computing the predictions accuracies of the *model*_*k*_ on these permuted subsets (*accuracy*_*k, perm*_). If the proportion of *accuracy*_*k, perm*_ ≥ *accuracy*_*k*_ over all *boot* comparisons is above a defined threshold (5% by default), *S*_*f*_ is declared not significant, and the next candidate feature *f*′ has the rank closest to the mean of *rank*(*h*) − 1 and *rank*(*f*) + 1, where *h* is the last significant feature detected. Otherwise *S*_*f*_ is significant, and the next *f*′ is the feature with the rank closest to the mean of *rank*(*f*) − 1 and *rank*(*l*) + 1 where *l* is the last non-significant feature detected. At the end of the half-interval search, the dataset is restricted to the features of ranks < *rank*(*f*_*ns*_). If no feature has been found significant, the dataset is restricted to the half of features with lowest ranks, but these features are not registered as significant in this round.
4. **Building the final model**. Steps 1–3 are repeated until, for a given round, candidate features are all found significant (these features then correspond to the signature), or until there is no feature left to be tested (the signature is empty). When the signature is not empty, the final model is then obtained by a single training on the dataset containing all observations and restricted to the features from the signature.

**Algorithm 1:** The *biosigner* algorithm. The inputs are **X**: *sample* × *feature* matrix of intensities; α: significance level; and *boot*: number of bootstraps.

1: **function** BIOSIGNER(**X**, α, *boot*)
2: **while** the algorithm has not converged and **X** contains features **do**
3: Create *boot* training sets *train*_*k*_ and test sets *test*_*k*_ by bootstrapping [step 1]
4: **for** each training set *train*_*k*_ **do**
5: Train the model *model*_*k*_
6: Compute the feature ranking *rank*_*k*_ according to the selected metric
7: Compute the balanced prediction accuracy *accuracy*_*k*_ using *test*_*k*_
8: **end for**
9: Compute the feature final ranking according to equation 1 [step 2]
10: Half-interval search for the largest non-significant feature subset *S* using α as the threshold rate [step 3]
11: Restrict **X** to the features not in *S*
12: **end while**
13: Repeat the **while** procedure above until either **X** is constant (i.e., the *S* subset from the last round was empty), or until **X** does not contain any feature left [step 4]
14: If **X** contains features, return the model trained on **X**
15: **end function**

## 3. Materials and methods

### 3.1. Datasets

- **LC-HRMS metabolomics**
  - *sacurine*: Urine from human adults
    The metabolomics analysis of urine samples from a cohort of employees from the CEA Saclay research institute by liquid chromatography coupled to high-resolution mass spectrometry (LC-HRMS) has been described previously (Roux et al., 2012; Thevenot et al., 2015). Briefly, the samples were analyzed by ultra-high performance liquid chromatography (Hypersil GOLD C18 column, Thermo Fisher) coupled to a high-resolution mass spectrometer (LTQ-Orbitrap Discovery, Thermo Fisher). The raw files were preprocessed by using the XCMS (Smith et al., 2006) and the CAMERA R packages (Kuhl et al., 2012). Annotation at levels 1 and 2 from the metabolomics standard initiative (MSI; Sumner et al., 2007) was performed by in-house and public databases query in addition to MS/MS experiments. Finally, the intensities of the annotated metabolites were validated by using the Quan Browser module from the Xcalibur software (Thermo Fisher), corrected for signal drift and batch-effect, and *log*_10_ transformed (Thevenot et al., 2015). Here, we used the *sacurine* subset corresponding to the negative ionization mode, which consists of 183 samples and 109 annotated metabolites, and is available from the *ropls* R Bioconductor package.
  - *spikedApples*: Apples spiked with known compounds
    One *control* group of 10 apples and several *spiked* sets of the same size have been analyzed by LC-HRMS (SYNAPT Q-TOF, Waters; Franceschi et al., 2012). The spiked mixtures consisted of 2 compounds which were not naturally present in the matrix, and 7 compounds aimed at achieving a final increase of 20–100% of the endogeneous concentrations. The dataset is included in the *BioMark* R Bioconductor package (Franceschi et al., 2012). The *control* and the first *spiked* groups (i.e., a total of 20 samples) were used in this study.
  - *diaplasma*: Plasma from diabetic patients
    Collection of plasma samples from type 1 and type 2 diabetic patients (Hôtel-Dieu, Paris, France) was performed with informed consent of the subjects, in accordance with the 1964 Helsinki declaration and its later amendments. Samples were analyzed by ultra-high performance liquid chromatography (Nexera, Shimadzu) coupled to a high resolution mass spectrometry operating in the negative ionization mode (Orbitrap Exactive, Thermo Fisher). Raw data were processed with XCMS (Smith et al., 2006) and CAMERA (Kuhl et al., 2012), and the resulting peak table was annotated by matching the measured m/z and retention times against an in-house database from MS spectra of pure compounds. Signal drift was corrected by using a loess fit of the intensities from quality control (*pool*) samples injected periodically (Dunn et al., 2011; Thevenot et al., 2015). Features which did not meet the following quality control criteria were discarded: (i) ratio of mean intensity in samples over mean intensity in blanks (mobile phase only) >2, (ii) significant correlation between the intensities of the diluted pool samples and the dilution factor, and (iii) coefficient of variation of pool intensities < 30%. Five samples with *p*-values < 0.001 by using either the Hotellings' *T*^2^ outlier test, or the Z-score corresponding to the highest deviation of intensity quantiles (Alonso et al., 2011) were removed. Finally, the intensities were *log*_10_ transformed and the few missing values (< 0.01%) were set to 0.
- **Microarray transcriptomics**
  - *leukemia*: Bone marrow from acute leukemia patients
    Samples from patients with acute lymphoblastic (ALL, 47 patients) or myeloid (AML, 25 patients) leukemia have been analyzed by using Aymetrix Hgu6800 chips. The resulting dataset contains expression data from 7129 gene probes (Golub et al., 1999), and is available from the *golubEsets* R Bioconductor package.

### 3.2. The *biosigner* algorithm

The principles of the algorithm are detailed in the *Theory* section and are illustrated in Figures 1, 2.

#### 3.2.1. Wrapped classifiers

The algorithm was independently wrapped around three machine learning approaches, namely Partial Least Squares—Discriminant Analysis (PLS-DA), Random Forest, and Support Vector Machines (SVM), by using the following implementations:

- **Partial Least Squares Discriminant Analysis (PLS-DA)**
  The PLS-DA implementation from the *ropls* bioconductor package (Thevenot et al., 2015; version 1.2.2) was used after mean-centering and unit-variance scaling of the features. Briefly, the binary response is converted to a numeric vector **y** of values in {-0.5; 0.5}, and a PLS regression is performed with the NIPALS algorithm (Wold et al., 2001; Barker and Rayens, 2003). The number of components is determined automatically as follows (Eriksson et al., 2001): a new component *h* is added to the model if :
  1. *R*2*Y*_*h*_ ≥ 1%, i.e., the percentage of **y** variance explained by component *h* is more than 1%, and
  2. *Q*2*Y*_*h*_ = 1 − *PRESS*_*h*_ ∕ *RSS*_*h*−1_ ≥ 0 (or 5% when the number of samples is less than 100), i.e., the predicted residual sum of squares (*PRESS*_*h*_) of the model including the new component *h* estimated by 7-fold cross-validation is less than the residual sum of squares (*RSS*_*h* − 1_) of the model with the previous components only (with *RSS*_0_ = (*n* − 1)*var*(**y**)).

Finally, the predictive performance of the full model is assessed by the cumulative *Q*2*Y* metric: $Q2Y=1-\overset{r}{\underset{h=1}{\prod }}\left(1-Q2Y_{h}\right)$. We have *Q*2*Y* ∈ [0, 1], and the higher the *Q*2*Y*, the better the performance. However, models trained on datasets with a larger number of features compared with the number of samples can be prone to overfitting: in that case, high *Q*2*Y* values are obtained by chance only. To estimate the significance of *Q*2*Y* (and *R*2*Y*) values, Szymanska et al. (2012) therefore proposed to perform permutation testing: models are built after random permutation of the **y** values, and *Q*2*Y*_*perm*_ are computed. The *p*-value is equal to the proportion of *Q*2*Y*_*perm*_ above *Q*2*Y* (in this study, the number of random permutations was set to 1000).

- **Random Forest**
  The implementation of the method from Breiman (2001) in the *randomForest* R package was used (Liaw and Wiener, 2002; version 4.6.10). The number of trees was set to 500, and the number of candidates randomly sampled at each split was the squared root of the total number of features.
- **Support Vector Machine (SVM)**
  The *e1071* R package (Meyer et al., 2014; version 1.6.4) implements the *libsvm* algorithm (Chang and Lin, 2011). Features were mean-centered and unit-variance scaled prior to linear SVM modeling with *cost* = 1.

#### 3.2.2. Resampling (step 1)

The default number of bootstraps was 50.

#### 3.2.3. Feature ranking (step 2)

The following metrics were used: the variable importance in projection (VIP) for PLS-DA (Wold et al., 2001), the variable importance based on the error rate for Random Forest (Breiman, 2001), and the squared weights for SVM (Guyon et al., 2002).

### 3.3. Quality of the feature selection

The (balanced) prediction *accuracy* of a classifier is the mean of *sensitivity* and *specificity*. The *stability* of the signature was determined as follows: the dataset was split into 10 subsets, each containing 90% of the samples, and the feature selection approach was applied to each subset, resulting in 10 signatures. The stability was the average similarity over all pairs of signatures. We used the similarity measure proposed by Lustgarten and colleagues, since (i) it is adjusted for the commonality of subsets obtained by chance only, and (ii) it allows to compare signatures of different sizes (Lustgarten et al., 2009). The performance-robustness trade-off (hereafter *performance*) was computed as the harmonic mean of *accuracy* and *stability* (Determan, 2015).

### 3.4. Compared feature selection methods

#### 3.4.1. Filter methods

Features were filtered according to their VIP value from PLS-DA, at a threshold of either 1 or 1.5.

#### 3.4.2. Wrapper methods

We implemented recursive feature elimination (RFE) for SVM as described in Guyon et al. (2002). We used a 50 bootstrap resampling strategy (identical to *biosigner*), and removed the 20% features with highest ranks at each iteration. The subset giving the best prediction accuracy was selected. The same approach was also implemented for PLS-DA and Random Forest. For each classifier, the machine learning parameters and feature ranking metrics are identical to those from the *biosigner* algorithm, as described above.

#### 3.4.3. Embedded methods

Prediction analysis for microarrays (PAM; also called nearest shrunken centroids), sparse PLS-DA, and Lasso/Elastic net were performed with the the *pamr* (Hastie et al., 2014), *spls* (Chung et al., 2013), and *glmnet* (Friedman et al., 2010) R packages, respectively.

### 3.5. Software

The *biosigner* package was written in R (R Core Development Team, 2015; version 3.2.2) and is available (http://bioconductor.org/packages/biosigner) from the Bioconductor repository (Gentleman et al., 2004). It includes the *diaplasma* LC-HRMS metabolomics dataset. The package was run on a laptop computer (Windows 7; Intel Core i5 2.6 GHz processor; 8 GB RAM). The *biosigner* algorithm is also available with a graphical interface, as a Galaxy module within the *Workflow4Metabolomics.org* online resource for computational metabolomics (http://workflow4metabolomics.org; Giacomoni et al., 2015).

## 4. Results

### 4.1. Development of the *biosigner* algorithm

We developed a new wrapper algorithm to select features which significantly improve the prediction of any binary classifier (see Section 2 and Figure 1). A feature subset *S*_*f*_ is declared significant if the predictions on test subsets (generated by resampling) are less accurate after randomly permuting the intensities of all features in *S*_*f*_ (Figure 2). The dataset is then restricted to the significant features and the procedure is iterated until the set of significant features remains unchanged (i.e., corresponds to the final signature), or until there is no feature left in the dataset to be tested (*Theory* section and Figure 1). The algorithm was wrapped around three machine learning approaches, namely Partial Least Squares Discriminant Analysis (PLS-DA), Random Forest, and Support Vector Machine (SVM), which rely on specific mathematical backgrounds (latent variables, decision trees, and kernel methods, respectively). For each classifier, the *biosigner* algorithm therefore returns a stable final *S* signature (possibly empty), in addition to several tiers (from *A* to *E*) corresponding to the features discarded during one of the previous iterations (e.g., features from the *A* tier were selected in all but the last iteration).

### 4.2. Evaluation on published metabolomics and transcriptomics datasets

**Figure 3:**
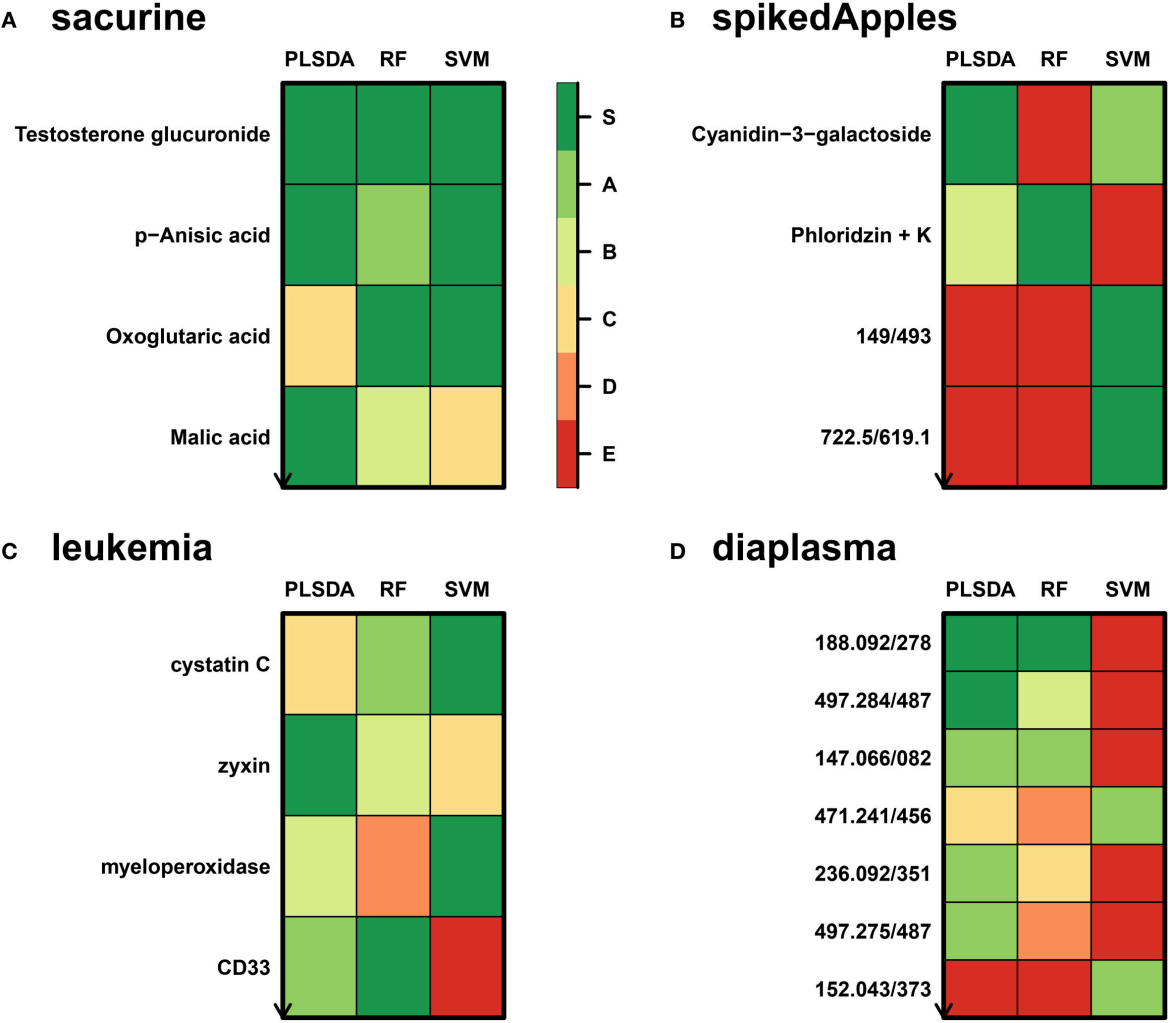
***biosigner* discovery of molecular signatures from biological and clinical omics datasets**. **(A,B,D)** The *sacurine, spikedApples*, and *diaplasma* metabolomics datasets result from the LC-HRMS analysis of, respectively, urines from male or female adult volunteers (Thevenot et al., 2015), control apples or apples spiked with a mixture of known compounds (Franceschi et al., 2012), and plasma from type 1 or 2 diabetic patients (see Section 3). **(C)** The *leukemia* transcriptomics dataset has been obtained by microarray analysis of bone marrow from patients with acute lymphoblastic or myeolid leukemia (Golub et al., 1999). The *biosigner* signatures of *gender* (*sacurine* dataset), *spiking* (*spikeApples*), *ALL/AML* leukemia (*leukemia*), and diabetes *type* (*diaplasma*) obtained independantly by each of the 3 classifiers are shown (for *diaplasma*, the extended signatures up to tier *A* are displayed). The *S* tier corresponds to the final signature, i.e., metabolites which passed all the selection iterations determining whether the candidate variables significantly improve the model prediction. In contrast, metabolites from the other tiers were discarded during all but the last (*A*) or previous (*B* to *E*) iterations. RF, Random Forest.

**Figure 4:**
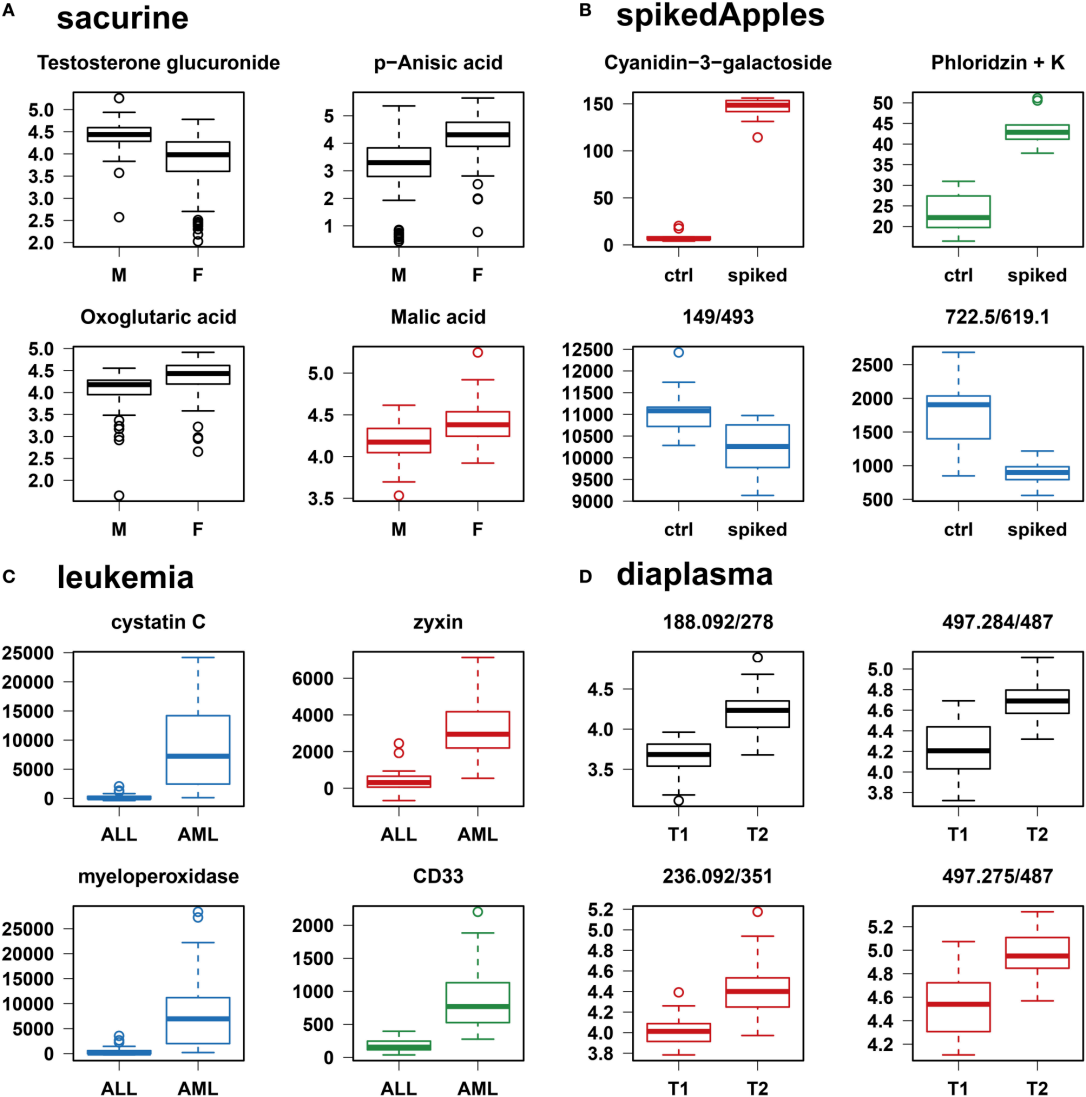
**Individual boxplots of the features selected by at least one of the classification methods**. **(A–C)** Features specifically selected by a single classifier are colored (*red* for PLS-DA, *green* for Random Forest, and *blue* for SVM). **(D)** For the *diaplasma* dataset, two representative features from the *A* PLS-DA tier are also displayed. M/F, male/female; ctrl, control; ALL/AML, acute lymphoblastic/myeloid leukemia; T1/T2, type 1/type 2 diabetes.

We addressed the performance of the *biosigner* algorithm by analyzing the signatures obtained on two metabolomics and one transcriptomics real datasets. We started with a well-annotated human metabolomics dataset, in which the concentrations of 109 metabolites have been measured in urine samples from a cohort of 183 adult volunteers by using liquid chromatography coupled to high-resolution mass spectrometry (LC-HRMS, Thevenot et al., 2015). A previous study of the physiological concentration differences between males and females using this *sacurine* dataset (Thevenot et al., 2015) has shown that: (i) no specific gender clusters were observed by Principal Component Analysis (PCA), (ii) 45 metabolites had a significant difference of medians between genders (with Mann-Whitney *U*-tests and a False Discorvery Rate threshold of 5%), and (iii) PLS-DA modeling of *gender* had a significant *Q*2*Y* value of 0.58 (metric between 0 and 1 estimating the prediction performance by cross-validation; see Section 3). By applying the *biosigner* algorithm, signatures consisting of 2 (Random Forest) or 3 (PLS-DA, SVM) metabolites (i.e., less than 3% of the initial features) were identified (Figure 3A). Testosterone glucuronide was common to all 3 signatures, and oxoglutaric acid and p-anisic acid to 2 of them. All selected metabolites had a clearcut difference of intensities between males and females (Figure 4A). Prediction accuracies of the models restricted to the signatures were all superior or equal to the models trained on the full dataset (Table 1).

**Table 1:** **Molecular signatures extracted by *biosigner* from metabolomics and transcriptomics biological and clinical datasets, and prediction accuracies of the corresponding models before and after feature selection**.

			***sacurine***	***spikedApples***	***leukemia***	***diaplasma***
**Systems level**			**Metabolomics**	**Metabolomics**	**Tanscriptomics**	**Metabolomics**
**Groups**			**male/female**	**control/spiked**	**ALL/AML**	**type 1/type 2**
Samples			183	20	72	69
Variables			109	1632	7129	5501
Sample/variable ratio			1.7	0.012	0.01	0.013
Time (min)			0.4	0.4	4.8	3.5
	PLS-DA		3	1	1	2
Signature S	Random forest		2	1	1	1
	SVM		3	2	2	0
	PLS-DA		5	2	2	5
Signature AS	Random forest		3	3	2	2
	SVM		8	20	5	2
		Full	0.87	0.81 (ns)	0.95	0.83
	PLS-DA	AS	0.88	1	0.88	0.9
		**S**	**0.89**	**1**	**0.87**	**0.91**
		Full	0.86	0.92	0.92	0.81
Accuracy	Random forest	AS	0.87	1	0.93	0.82
		**S**	**0.86**	**0.99**	**0.92**	**0.81**
		Full	0.88	0.84	0.93	0.83
	SVM	AS	0.88	0.97	0.94	0.69
		**S**	**0.89**	**0.86**	**0.95**	**na**

*The accuracies of the models trained on the final S signatures are in bold. na: for the diaplasma dataset, no feature was selected as significant in the last iteration with the SVM classifier; ns: the Q2Y value of the PLS-DA classifier on the full spikedApples dataset is not significant indicating that the model before feature selection is overfitted*.

We then studied a recently published metabolomics dataset spiked with known compounds (Franceschi et al., 2012). The *spikedApples* dataset results from the LC-HRMS analysis of groups of apples which have been spiked with various concentrations of 7 endogenous metabolites and 2 exogenous compounds. The peak table used in this study consists of two groups of 10 apple samples (*control* and *spiked*), and 1632 features (among which 22 were identified by the authors as originating from the spiked molecules; Franceschi et al., 2012). Preliminary modeling of the *control* vs. *spiked* response by PLS-DA with the full dataset indicated that the *Q*2*Y* was not significant: feature selection was therefore mandatory to avoid overfitting (Table 1). The *biosigner* algorithm identified complementary signatures of 1 or 2 features (Figure 3B). Classifiers trained on the dataset restricted to the signatures outperformed the models trained on the full dataset. Interestingly, the single features selected by PLS-DA and Random Forest corresponded to cyanidin-3-galactoside and a potassium adduct of phloridzin, respectively, which both belong to the list of expected discriminating metabolites: the former is absent from the natural matrix (*control* group), and the concentration of the latter is increased up to 80% in the *spiked* group (Franceschi et al., 2012; Figure 4B). Surprisingly, the two features selected by SVM are less concentrated in the *spiked* group (Figure 4B). It should be noted, however, that such a decrease in concentrations was found significant by univariate hypothesis testing for a total of 9 features (including 722.5/619.1), compared with 17 features with significantly increased concentrations. The high proportion (35%) of features with decreased concentrations among the discriminating signals may therefore explain why two of them have been included in the SVM classification rule.

Finally, to demonstrate that our approach can be applied to other omics data, we analyzed the reference transcriptomics dataset resulting from the microarray analysis of bone marrow samples from 72 leukemia patients (Golub et al., 1999). Preliminary univariate hypothesis testing indicated that 1154 out of the 7129 features were significant for median difference between the lymphoblastic and myeloid groups. In addition, the two groups were clearly visible on the score plots from PLS-DA, but not PCA (data not shown). Our algorithm identified signatures from 1 to 2 gene probes (Figures 3C, 4C). Random Forest and SVM (but not PLS-DA) models trained on the signatures had superior or equal prediction accuracies than the classifiers trained on the full dataset (Table 1). The four selected features ranked 1st, 5th, 7th, and 12th in the list of significant variables by univariate hypothesis testing, ordered by increasing *p*-values. Three of them, cystatin C, zyxin, and CD33, were also part of the 50 gene signature selected by Golub and colleagues on the basis of a filter metric derived from the Student's statistic (Golub et al., 1999).

### 4.3. Application to the discovery of signatures discriminating type 1 and type 2 diabetic patients

We applied our methodology to the study of metabolomics signatures between type 1 and type 2 diabetes mellitus. Plasma samples from 69 diabetic patients were analyzed by LC-HRMS, and a peak table containing 5501 features was obtained after file preprocessing (see Section 3). Seven hundred features were found significant by univariate hypothesis testing. It should be noted that, because type 2 patients were significantly older than type 1 individuals in this cohort (as in the general population of diabetic patients), some of the observed variations may be the result of physiological aging (see Section 5). Unsupervised analysis by PCA did not evidence any clustering according to diabetic *type*, in contrast to PLS-DA modeling which resulted in a significant *Q*2*Y* value of 0.46. By further applying the *biosigner* algorithm, signatures of 1 and 2 features were obtained with the PLS-DA and Random Forest classifiers, respectively (Figure 3D), in an average computation time of 3.4 min pro classifier on a laptop computer. The two features were highly significant by hypothesis testing of difference between *type* medians (*p* < 10^−7^ and *p* < 10^−6^, respectively; Figure 4D), and to a lesser extent by testing of correlation with *age* (*p* < 10^−4^ and *p* < 10^−3^) or *body mass index* (*p* < 10^−2^ and *p* < 5 × 10^−2^). Surprisingly, the *S* signature from SVM was empty (i.e., no feature was selected as relevant in the last extraction round). We therefore investigated the features from the antepenultimate (*A*) tier (Figure 3D): variables from the *A* SVM signature were distinct from PLS-DA and Random Forest, and the accuracy of the SVM model restricted to the *A* signature decreased (from 83% to 69%; Table 1). In contrast, accuracies of the PLS-DA and Random Forest models restricted to the *S* signatures (91% and 81%, respectively) were both superior or equal to the models trained on the full dataset (83% and 81%).

### 4.4. Stability of the signatures and sensitivity/specificity of the selection

The influence of bootstraping on the *stability* of the *S* and *S+A* signatures was assessed for each of the 4 datasets by increasing the number of bootstraps from 5 to 200, and looking for differences in stability by using repeated measure ANOVA. No significant difference was observed above 20 bootstraps for PLS-DA and SVM, and 10 bootstraps for Random Forest. The number of 50 bootstraps was thus selected as the default value in *biosigner*, and used in all computations.

To assess the *sensitivity* and the *specificity* of the methodology, datasets containing known discriminant variables were simulated (see Supplementary Material). To avoid making hypotheses about the structure of an “omics” dataset (e.g., noise, intensity distribution, etc.), we started from the real metabolomics and transcriptomics datasets and removed all features which were significant by univariate hypothesis testing (Wehrens and Franceschi, 2012). We then increased the discriminant capacity of one of the features by multiplying the intensities in one of the sample groups by a factor. The factor was chosen so that the target feature was still not detected at a False Discovery Rate of 0.05. By applying this methodology to our transcriptomics and metabolomics datasets, we observed that, despite the high ratio of variables to samples (up to 100 for the *leukemia* dataset), the target feature was detected with a high sensitivity (from 60% up to 100% in the union of the three classifier signatures) and a high specificity (more than 98%; see Supplementary Material).

### 4.5. Comparison with alternative feature selection methods

**Figure 5:**
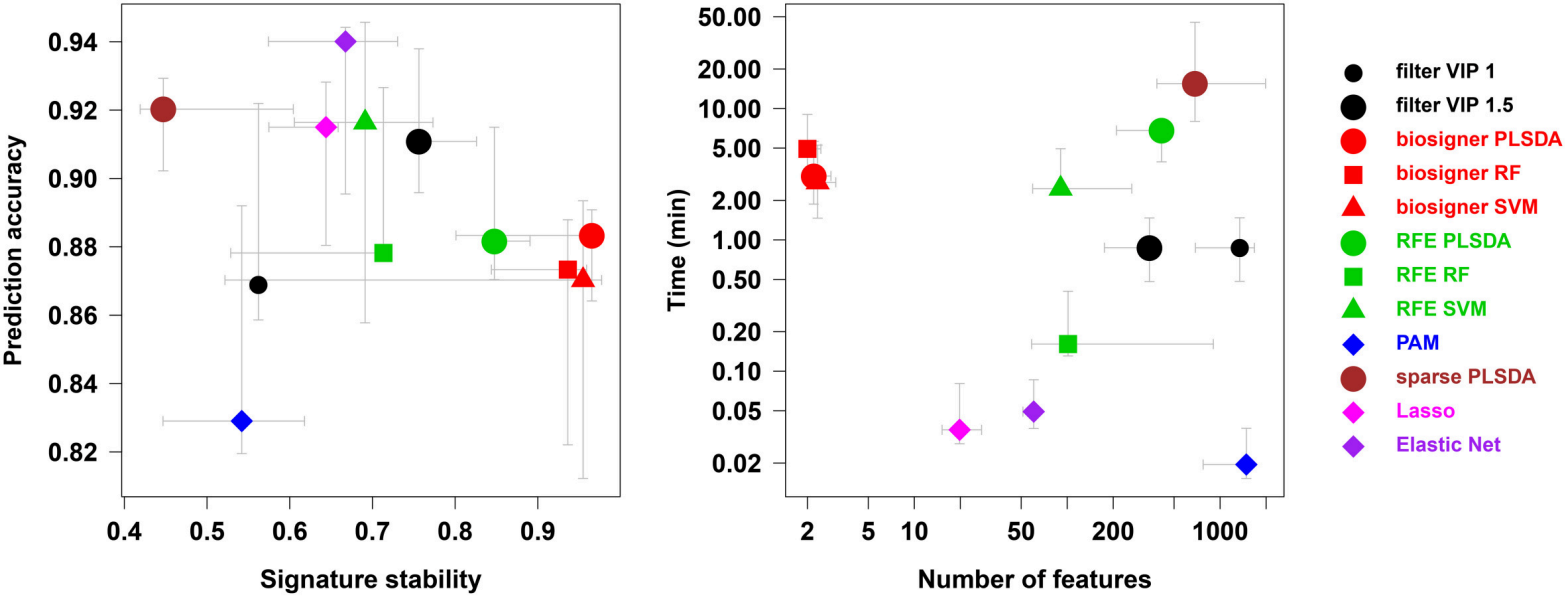
**Comparison of *biosigner* with alternative feature selection approaches**. Metric values are from Table 2: the median and interquartile range for the *sacurine, leukemia*, and *diaplasma* datasets are indicated as colored symbols and grays arrows, respectively. PAM: prediction analysis of microarrays (also called nearest shrunken centroids); RF, Random Forest; RFE, recursive feature elimination; filter VIP, filtering by the *variable importance in projection* metric from PLS-DA.

We compared our algorithm with 6 alternative approaches for feature selection, namely VIP filtering (at the 1 and 1.5 thresholds; Mehmood et al., 2012), recursive feature elimination wrapper (RFE; Guyon et al., 2002), and 4 embedded methods (prediction analysis of microarray, PAM, also called nearest shrunken centroids, Tibshirani et al., 2002, sparse PLS-DA, Chun and Keles, 2010, Lasso, Tibshirani, 1996, and Elastic Net, Zou and Hastie, 2005). In particular, to achieve a comprehensive comparison between the *biosigner* and RFE wrapper approaches, we applied the RFE methodology not only to SVM (as initially described, Guyon et al., 2002) but also to PLS-DA and Random Forest. For each algorithm, the *accuracy* of the final model, the *size* and *stability* of the signature, as well as the *performance* (harmonic mean between *accuracy* and *stability*) were computed for each of the 4 datasets, and the running *time* on a laptop computer was recorded (Table 2). The best *performances* were achieved by using the *biosigner* algorithm: whereas higher prediction *accuracies* could be obtained by regularized methods such as Elastic Net, the signatures from *biosigner* were usually more stable. Surprisingly, the stability of the Random Forest and SVM *biosigner* signatures on the *spikedApples* dataset (and also of the SVM signature on the *diaplasma* dataset) was low. The variability observed with the *spikedApples* dataset may be due to the small number of samples (10 in each group) in addition to the very small proportion of discriminating signals. The median and interquartile metric values for the *sacurine, leukemia* and *diaplasma* datasets are plotted on Figure 5. We see that *biosigner* selects restricted signatures, which are usually of high stability and provide high prediction accuracy.

**Table 2:** **Comparison of *biosigner* with alternative feature selection methods**.

		**Accuracy**	**Stability**	**Performance**	**Number of features**	**Time (min)**
		sacu spik leuk diap	sacu spik leuk diap	sacu spik leuk diap	sacu spik leuk diap	sacu spik leuk diap
VIP filter	≥1	0.87 **1.00 0.97** 0.85	0.56 0.50 0.54 0.57	0.68 0.67 0.70 0.68	36 332 2012 1344	0.1 0.3 2.1 0.9
	≥1.5	0.88 **1.00** 0.96 **0.91**	0.90 0.70 0.76 0.73	0.89 0.83 0.85 0.81	5 72 418 345	0.1 0.4 2.1 0.9
	PLS	0.88 **1.00** 0.84 0.90	0.97 **1.00** 0.64 **0.98**	0.92 **1.00** 0.73 **0.94**	4 **1 2 2**	0.7 1.0 8.2 3.1
*biosigner*	RF	0.87 0.90 0.90 0.77	**0.98** 0.11 0.75 0.94	**0.92** 0.20 0.82 0.85	**2 1 2** 3	1.0 0.8 13.1 4.9
	SVM	0.87 0.67 0.92 0.75	0.95 0.07 **1.00** 0.09	0.91 0.12 **0.96** 0.16	4 1 2 2	0.2 0.7 7.8 2.7
	PLS	0.86 0.90 0.95 0.88	0.93 0.43 0.85 0.83	0.89 0.58 0.90 0.86	6 539 480 414	6.8 2.0 7.8 1.0
RFE	RF	0.88 0.95 **0.97** 0.88	0.72 0.17 0.34 0.71	0.79 0.28 0.51 0.79	16 566 1705 101	0.7 **0.0** 0.2 0.1
	SVM	0.92 0.95 **0.97** 0.80	0.52 0.90 0.86 0.69	0.66 0.92 0.91 0.74	28 41 438 91	2.5 1.1 7.4 2.4
PAM		0.83 **1.00** 0.95 0.81	0.35 **1.00** 0.69 0.54	0.49 **1.00** 0.80 0.65	64 **1** 1485 1575	**0.0 0.0 0.1 0.0**
sPLS		0.92 **1.00** 0.94 0.88	0.45 0.80 0.39 0.76	0.60 0.89 0.55 0.82	87 131 3280 685	0.5 1.6 75.3 15.5
Lasso		**0.94 1.00** 0.92 0.85	0.51 **1.00** 0.67 0.64	0.66 **1.00** 0.78 0.73	35 **1** 20 11	**0.0 0.0 0.1 0.0**
Elast. Net		0.94 **1.00** 0.95 0.85	0.48 1.00 0.79 0.67	0.64 1.00 0.86 0.75	42 2 60 69	**0.0 0.0 0.1 0.0**

*The metrics obtained with the sacurine (sacu), spikedApples (spik), leukemia (leuk), and diaplasma (diap) datasets are shown (performance is the harmonic mean between the accuracy and the stability). For each metric and each dataset, the method(s) with optimum value is/are in bold. Elast. Net, Elastic Net; PAM, prediction analysis of microarrays (also called nearest shrunken centroids); RF, Random Forest; RFE, recursive feature elimination; sPLS, sparse PLS-DA; VIP filter, filtering by the variable of importance in projection metric from PLS-DA*.

### 4.6. Implementations of the *biosigner* methodology into an R/Bioconductor package and a Galaxy/Workflow4metabolomics module

To share the algorithm and its source code with the bioinformatics community, a *biosigner* R package (http://bioconductor.org/packages/biosigner) was published on the Bioconductor repository (Gentleman et al., 2004). Furthermore, to provide a graphical interface for the experimenter community, we developed a Galaxy module which was integrated into the Worfklow4Metabolomics (W4M) online infrastructure for computational metabolomics (http://workflow4metabolomics.org; Giacomoni et al., 2015). The full *history* (i.e., workflow and associated input and output data) of the statistical analysis of the *diaplasma* dataset described in this study is publicly available on W4M, with the W4M00003 reference number.

## 5. Discussion

We have developed a new algorithm for feature selection, named *biosigner*, which iteratively removes subsets of features that do not significantly improve the prediction accuracy of the model. Compared with alternative wrapper approaches (such as recursive feature elimination) based on the prediction accuracy only, *biosigner* selects feature subsets which *significantly* improve this prediction. The two main innovations of the algorithm are: (1) the significance of a feature subset is estimated by comparing the model predictions before and after random permutation of the intensities of these features in test subsets generated by resampling, and (2) the whole feature selection procedure is repeated recursively until all features of the selected subset are found significant, or until there is no feature left to be tested. Since permutations occur only in the test subsets, the number of models to be built is limited and the algorithm is fast (only a few minutes on a laptop for datasets of several thousands of variables). *biosigner* returns (i) the final *S* signature, (ii) the tiers (*A* to *E*) containing the features discarded during one of the previous selection rounds, and iii) the models trained on the *S* (and *S+A*) signatures to be used for future predictions.

The term *significance* is usually associated in the statistical literature to a hypothesis test and to a so called *null* hypothesis. Here, we do not assess the significance of the model itself, but we rather estimate the influence of a given subset of features on the prediction accuracy of the model. However, since the number of selected features in the final signature is restricted (i.e., smaller than the number of samples), the risk of overfitting is limited. Also, since the training and testing subsets are not set apart during the full procedure (because of the resampling between the selection rounds), the returned performance of the final model may be slightly over-optimistic. External validation on a new dataset is therefore required to refine the estimation of the model accuracy (Esbensen and Geladi, 2010).

By wrapping the *biosigner* algorithm around 3 binary classifiers with specific mathematical backgrounds (PLS-DA, Random Forest, and SVM), we observed on three published metabolomics and transcriptomics datasets that the signatures had some degree of similarity (e.g., at least one of the features in the tiers *S+A* was common to at least two classifiers), but also included classifier-specific features. For example, with the *spikedApples* dataset, SVM selects features with opposite variations compared with PLS-DA and Random Forest. The complementarity between the signatures is in agreement with several recent studies reporting classifier-specific results depending on the structure of the dataset (correlation between features, noise, proportion of zeros intensities; Christin et al., 2013; Tarca et al., 2013; Determan, 2015). The discrepancies come from the specific weights each classifier assigns the variables (or the samples in the case of SVM). In fact, if the same ranking metric is used for all classifiers in our algorithm, signatures become more similar (see Supplementary Material). The use of classifier-specific metrics in *biosigner* (i.e., VIP for PLS-DA, variable importance for Random Forest, and squared weights for SVM) should therefore increase the chances of discovering distinct features of interest.

The signatures obtained with the three datasets were short (up to 3 features), and the restriction to these signatures usually resulted in higher prediction accuracies of the classifiers (except for the PLS-DA model on the *leukemia* dataset). Importantly, restricting to small signatures also helped avoiding the risk of overfitting: for instance, the high performance of PLS-DA model of the *spikedApples* full dataset was not significant, contrary to the model built on the single feature signature.

The selected molecules were also shown to be in good agreement with the reported results: the 2 features from the *spikedApples* PLS-DA and Random Forest signatures were related to the spiked compounds (Franceschi et al., 2012), and the 3 gene probes selected by PLS-DA and SVM on *leukemia* dataset were already included in the published signature (Golub et al., 1999). Interestingly, the probe selected by Random Forest, myeloperoxidase, is a cytochemical marker for the diagnosis (and also potentially the prognosis) of acute myeloid leukemia (AML; Matsuo et al., 2003). Although myeloperoxidase would also have been included in the published signature if the full dataset had been used for training (as in this study) instead of the 38 sample subset (Golub et al., 1999), this feature would have been ranked only in the 24th position. Altogether, the above results show that *biosigner* selects relevant signatures providing high and significant prediction accuracy of the classifiers.

Comparison with alternative feature selection strategies revealed that *biosigner* specifically selected stable signatures. In contrast, Lasso and Elastic net classifiers showed higher accuracy but the signatures were more prone to instability, in agreement with a previous analysis of 4 transcriptomics datasets (Haury et al., 2011): such methods can therefore be of high interest when classification accuracy (instead of feature selection) is the primary goal of the study. For feature selection, however, stability is critical since the subsequent validation steps leading to the diagnostic product will focus on the selected features only. Surprisingly, the stabilities of the *biosigner* SVM signatures were low for two of the datasets (*diaplasma* and *spikedApples*). This suggests that the combination of backward selection and feature ranking with the SVM square weights may result with some datasets in the elimination of relevant features during the first selection rounds.

The *biosigner* signatures were of restricted size. Such small signatures are pivotal for diagnosis purposes, where only a limited number of candidates are expected to enter the validation phase. The size of the signatures may be a consequence of the stringency of our selection algorithm, but also of the structure of the datasets analyzed in this study, where a very few variables are sufficient to efficiently discriminate between the two sample groups. It should be noted that the criterion for feature selection focuses on the added value of the tested features for model performance. Hence, additional features with equal relevance for prediction may not appear in the final *S* signature. As an example, only a fraction of the features related to the spiked compounds in the *spikedApples* dataset is selected. If an extended view of discriminating candidates is required, it may be of interest to look also at the *S+A* signature. An alternative is to re-run the *biosigner* algorithm after discarding the *S* signature, or after increasing the value of the α significance threshold.

We applied our methodology to address a new clinical question, namely the discovery of metabolomics signatures between type 1 and type 2 diabetic patients. The etiology of diabetes is complex (in particular the type 2, or insulino-resistant, form), and new biomarkers are needed for prognosis and diagnosis of the disease (Roberts et al., 2014). We thus performed a metabolomics analysis of plasma samples from diabetic patients by LC-HRMS. In this cohort, type 2 patients are significantly older (*p* < 10^−8^) and, to a lesser extent, have a higher *body mass index* (*p* < 10^−7^) than type 1 patients. Matching (e.g., by *age*), however, would have resulted in a very restricted subset of only 14 patients, in which no type 2 vs. type 1 significant feature could be found by univariate testing nor *biosigner* feature selection. We therefore used in this study the full *diaplasma* dataset instead (63 samples and 5501 features). Two features were selected either in the PLS-DA or Random Forest signatures. Importantly, when these features were modeled by a combination of the 3 covariates (diabetic *type, age*, and *body mass index*), only the *type* effect was found significant by analysis of variance, thus emphasizing the putative value of these markers in the classification of diabetes. Interestingly, one of the features from the *S+A* PLS-DA signature (497.275/487) matched with an isotope of a taurochenodeoxycholic acid fragment (according to m/z ratio and retention time): diabetes-associated changes in bile acid metabolism have been reported (Prawitt et al., 2011), and variation of the taurochenodeoxycholic acid concentration has very recently been described in urine of type 2 patients (Taylor et al., 2014). The *biosigner* signature, which requires further validation by MS/MS experiments and confirmation in another cohort study where patients and controls are matched by *age*, may therefore highlight new candidates for diabetes screening and diagnosis.

The *biosigner* algorithm is available as an R/Bioconductor package. As no clinical metabolomics dataset is currently available on Bioconductor to the best of our knowledge, we included the *diaplasma* LC-HRMS dataset into the package: this dataset should be useful for the benchmarking of new statistical and annotation algorithms. We also developed a Galaxy module which was integrated into the Workflow4metabolomics online infrastructure for computational metabolomics (W4M; Giacomoni et al., 2015). Galaxy is a powerful open-source workflow manager enabling users to build their own workflow by selecting the tools and the parameter values via a graphical interface (Goecks et al., 2010). Workflows and associated data inputs and outputs can be saved and shared, allowing fine tuning of parameters and reproducible research (Boekel et al., 2015). The W4M infrastructure is therefore of high interest for experimenters to build, run, and reference reproducible LC-MS, GC-MS, and NMR workflows, for developers to compare and diffuse their tools, and also for teachers to organize hands-on sessions (since no software installation is required).

In conclusion, the *biosigner* algorithm and the associated software tools should be of high value for biologists, practitioners, and biostatisticians, to identify robust biomarker signatures from large omics datasets for the development of new diagnostics.

## Author contributions

PR developed the algorithm and co-wrote the article. ET implemented the R package and the Galaxy module, worked on the application of the method to metabolomics, preprocessed the *diaplasma* dataset, and co-wrote the article. LC-HRMS analysis and annotation of the plasma samples from the *diaplasma* dataset were performed by SB and CJ.

## Funding

This work was supported by the Agence Nationale de la Recherche (ET: MetaboHUB national infrastructure for metabolomics and fluxomics, ANR-11-INBS-0010 grant) and the Seventh Framework Programme (PR; Biomargin project, grant agreement No 305499).

### Conflict of interest statement

The authors declare that the research was conducted in the absence of any commercial or financial relationships that could be construed as a potential conflict of interest.
