# Supplementary material for: biosigner: A New Method for the Discovery of Significant Molecular Signatures from Omics Data
Source: Front Mol Biosci. 2016 Jun 21;3:26. doi: 10.3389/fmolb.2016.00026 (PMC4914951; doi:10.3389/fmolb.2016.00026)
Supplement: Supplementary file 1 [file DataSheet1.pdf]

Supplementary Material: *biosigner*: A New Method for the Discovery of Significant Molecular Signatures from Omics Data

Philippe Rinaudo, Samia Boudah, Christophe Junot, and Etienne A. Thévenot

*Correspondence:
Etienne A. Thévenot
etienne.thevenot@cea.fr

1 INFLUENCE OF THE RANKING METRIC ON THE FINAL SIGNATURE

In *biosigner*, variables are ranked according to a metric which is specific to each classifier: variable importance in projection (VIP) for PLS-DA, variable importance for Random Forest, and squared weights for SVM. To study the influence of the ranking metric on the final signature, we used the same ranking metric for all classifiers (Figure 1). We observe that, whatever the dataset, the similarity between the classifier signatures is increased.

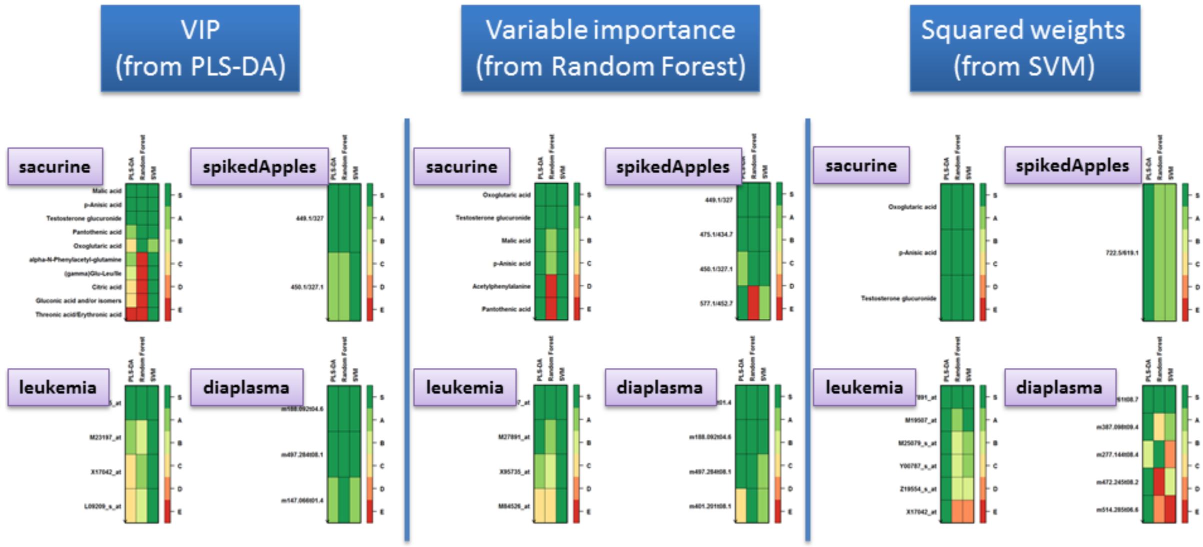

Figure 1. Signatures obtained with the *sacurine*, *spikedApples*, *leukemia*, and *diaplasma* datasets when the same ranking metric (indicated in blue on the top row) is used for all classifiers.

2 SENSITIVITY AND SPECIFICITY OF BIOSIGNER EVALUATED ON SIMULATED DATA

To simulate a dataset whose structure is close to real 'omic' data, we used the following procedure (adapted from Wehrens and Franceschi, 2012). Starting with a real dataset **X** (e.g. *diaplasma*), and a response factor **y** (e.g. diabetic *type*):

1. Generate a dataset with no discrimiant variable:
   a. Samples: *(optional)* Balance the number of samples in each class (by restricting the number of samples in each class to the minimum of class sizes),
   b. Variables: Remove all features which are significant by univariate hypthesis testing of difference of means between the two **y** classes,
   c. Check that the *biosigner* signature is empty.
2. Check if added discriminant variables are found in the *biosigner* signatures:
   a. Choose one (or more) variable(s) at random,
   b. For each selected variable, multiply all intensities in the sample class with the highest mean by a factor so that the p-value for the Student's t-test (after correction for multiple testing) will be above the usual significant threshold of 0.05 (e.g., 0.06),[1]
   c. Check if the discriminant variable(s) is/are found by *biosigner*,
   d. Repeat the procedure with another (set of) variable(s).

This methodology results in datasets with one (or several) *target* feature(s) whose discriminant capacity has been increased, but which is still not detected by univariate hypothesis testing at a False Discovery Rate of 0.05.

We applied the above approach to the four real transcriptomics and metabolomics datasets (Figure 2). For each simulation, a *sensitivity* (respectively 1 - *specificity*) was computed as 1 (or 0) if the target feature was present (or absent) in the signature (respectively as the number of other features in the signature divided by the total number of non-target features in the dataset). Despite the high ratio of variables to samples (up to 100 for the *leukemia* dataset), the target feature was detected with a high sensitivity (from 60% up to 100% in the *complete* signature) and a high specificity (more than 98%; Figure 2). Such a result, which should be confirmed with other simulation approaches, underlines the usefulness of *biosigner* for detecting discriminant features.

3 REFERENCES

Wehrens R. and Franceschi P. (2012). Meta-statistics for variable selection: The R package BioMark. *Journal of Statistical Software*, **51**.

[1] In the publication by Wehrens and Franceschi (2012), the values of the multiplicative factor are selected in advance (e.g., 1.5, 1.75).

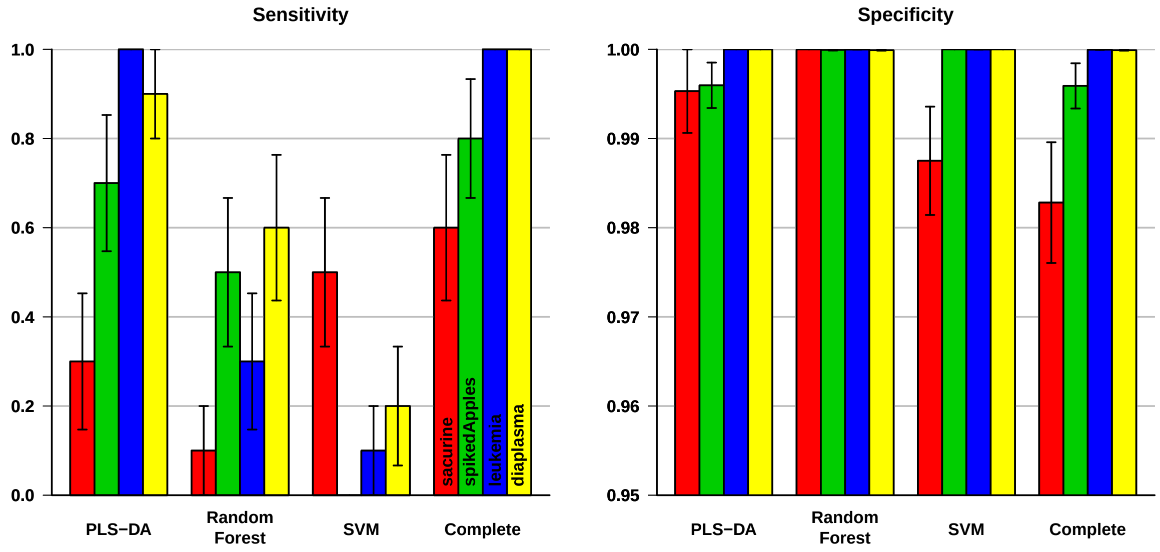

Figure 2. Sensitivity and specificity of the signatures assessed by simulation. After removing all significant variables by univariate hypothesis testing from the original datasets, the discriminant value of one feature was artificially increased by multiplying its intensities in one of the sample groups by a factor. The factor was chosen so that the modified feature remains undetected by univariate testing at a False Discovery Rate of 0.05. The average of the *sensitivity* (left) and *specificity* (right) after 10 simulations are shown. The bars correspond to the standard error of the mean. The *complete* signature is the union of the signatures provided by the three classifiers. The ratio between the number of variables and the number of samples is 0.39, 76, 100, and 94 for the *sacurine*, *spikedApples*, *leukemia*, and *diaplasma* datasets, respectively.
